# Thermodynamic Formation Properties of Point Defects in Germanium Crystal

**DOI:** 10.3390/ma15114026

**Published:** 2022-06-06

**Authors:** Jinping Luo, Chenyang Zhou, Qihang Li, Lijun Liu

**Affiliations:** School of Energy and Power Engineering, Xi’an Jiaotong University, Xi’an 710049, China; ivy.zhou@stu.xjtu.edu.cn (C.Z.); modaoshi532@stu.xjtu.edu.cn (Q.L.)

**Keywords:** germanium, point defects, formation free energy, formation entropy, molecular dynamics simulation, thermodynamic integration

## Abstract

Point defects are crucial in determining the quality of germanium crystals. A quantitative understanding of the thermodynamic formation properties of the point defects is necessary for the subsequent control of the defect formation during crystal growth. Here, molecular dynamics simulations were employed to investigate the formation energies, total formation free energies and formation entropies of the point defects in a germanium crystal. As far as we know, this is the first time that the total formation free energies of point defects in a germanium crystal have been reported in the literature. We found that the formation energies increased slightly with temperature. The formation free energies decreased significantly with an increase in temperature due to the increase in entropy. The estimated total formation free energies at the melting temperature are ~1.3 eV for self-interstitial and ~0.75 eV for vacancy, corresponding to a formation entropy of ~15 *k_B_* for both types of point defects.

## 1. Introduction

Germanium, as a semiconductor material, has excellent electrical, optical and structural characteristics. However, unlike silicon, germanium has attracted relatively less attention among the academic community and in industry. It has more than twice the mobility of charge carriers in comparison with silicon. This property makes it favorable to be used in the fabrication of high-speed electronic devices, e.g., memory cells. The dislocation-free germanium crystals are used as substrates for epitaxial structures when creating radiation-resistant power MOSFET-transistors for photoelectric converters and nanoscale transistor structures [1,2]. All of the various applications lead to high demands on the quality of crystals and, in particular, on the grown-in defects. The Czochralski (CZ)-grown germanium crystals usually inevitably include defects, which would influence the yield and performance of electronic devices built on Ge substrates [3]. The native point defects are the most fundamental lattice defects in the crystal, which can form voids, clusters and other complexes through diffusion and combination. A quantitative understanding and the subsequent control of the point defect formation in germanium crystals are therefore fundamentally important from the perspectives of both theory and technology. However, having an accurate knowledge of these atomic-scale phenomena is usually challenging due to the lack of reliable experimental data [4]. The classical molecular dynamics (MD) simulations are an alternative to provide useful information for this task. In comparison with calculations based on density functional theory (DFT), classical MD based on empirical potentials is less accurate but can provide large-scale and long-time simulations. In particular, thermo-physical properties related to free energies that require sampling over different configurations across the phase space can hardly be estimated through DFT.

The migration and formation energies of point defects in germanium crystals have been studied by Śpiewak et al. [3] and Kang et al. [5] based on atomic simulations. Śpiewak et al. calculated the vacancy and self-interstitial migration and formation energies using MD simulations based on several empirical potentials and compared their results to DFT and experimental values. They concluded that the Stillinger–Webber (SW) potential with the parameters of Wang et al. [6] can provide experimental comparable formation energies of neutral vacancy in germanium. The formation free energies of point defects in germanium crystal, however, are much less reported in previous research. As the temperature rises, the entropy contribution to the free energy is no longer ignorable. There may be some new features appearing driven by the entropy, i.e., Cowern et al. [7] suggested that there is a normal to “morph” structure transition of point defects in germanium from low to high temperature. The “morph” structure has a much higher configurational entropy, i.e., 30 *k_B_*. Unlike the perfect crystal whose free energy can be calculated easily using the Einstein crystal (EC) method [8], the estimation of defective crystal formation free energies is a more complex task. The EC method cannot work for the crystal with defects due to the mobility of the point defects, which will cause convergence problems at the end point of the thermodynamic integration (TI) [9,10]. Chiesa et al. [9] calculated the formation free energies of interstitial defect in bcc iron, where they took the harmonic crystal as the reference state in the TI and encountered severe convergence problems. They simply extrapolated the integration curve to the end point, which may bring in unexpected errors. Cheng and Ceriotti [10] calculated the vacancy formation free energy in bcc iron where they used two steps to obtain the high-temperature free energies. They first calculated the system free energy at a very low temperature, i.e., 100 K, to circumvent the above-mentioned diffusion problem, and then performed temperature-dependent thermodynamic integrations to obtain the desired free energy at a high temperature. Considering the above difficulties, people either use (formation) potential energies to replace the formation free energies or employ harmonic approximation (HA) to account for the entropic part.

In this research, we used MD simulations to calculate the formation free energies of point defects in germanium crystal based on our recently proposed method [11,12]. Wherever possible, the calculated results are compared with existing experimental or theoretical values. The remainder of the article is organized as follows: Section 2 introduces the methodology, which includes the description of the employed empirical potentials, the thermodynamic integration pathways and the simulation details. Results are shown in Section 3, while Section 4 presents the conclusions.

## 2. Methodology

### 2.1. Interatomic Potential Models for Germanium

We employed two empirical interatomic potential models: SW [6] and Tersoff [13] to describe the interaction between geranium atoms. These two potential models are very popular due to their relative simplicity and are widely applied to describe the covalent bonding of semiconductors such as: Si-Si, [14] Si-C, [15] Ga-N, [16], etc. However, these two potentials are very different, i.e., SW predicts the melting temperature *T_m_* of germanium to be 1487 K, which is close to the experimental value of 1210 K, while Tersoff overestimates *T_m_* to be 2737 K. This is a widely known weakness of the Tersoff potential; it also overestimates the *T_m_* of a silicon crystal. Considering the very large temperature difference, we used the scaled temperature *T*/*T_m_* in the results to make comparisons between different potentials as in our previous research [17,18]. There are several parameterizations for SW potential to model germanium. The comparisons between different potentials are reported in Refs. [3,19]. Here, we adopted the parameter set of Wang et al. [6] for SW, which is reported to predict experimental comparable formation energies of point defects in germanium crystal. The Tersoff potential can provide accurate cohesive energy and lattice parameters for germanium, silicon and their alloy systems [19]. In the following, we simply describe the format of the potential functions.

(a) SW potential

Within the SW potential, the overall potential energy U of the system is given by

(1) $$U=\sum_{i}\sum_{j>i}\phi_{2}\left(r_{ij}\right)+\sum_{i}\sum_{j\neq i}\sum_{k>j}\phi_{3}\left(r_{ij},r_{ik},\theta_{ijk}\right)$$

where $\phi_{2}$ and $\phi_{3}$ represent the two-body and three-body interactions between the atoms. $r_{ij}$ is the atom distance between atom *i* and *j*, and $\theta_{ijk}$ is the bond angle subtended at the central atom *i* between the bonds *ij* and *ik*. $\phi_{2}$ and $\phi_{3}$ take the form of:

(2) $$\phi_{2}\left(r_{ij}\right)=A\varepsilon \left[B{\left(\frac{\sigma }{r_{ij}}\right)}^{p}-{\left(\frac{\sigma }{r_{ij}}\right)}^{q}\right]\exp\left(\frac{\sigma }{r_{ij}-a\sigma }\right)$$

(3) $$\phi_{3}\left(r_{ij},r_{ik},\theta_{ijk}\right)=\lambda \varepsilon {\left[\cos\theta_{ijk}-h\right]}^{2}\exp\left(\frac{\gamma \sigma }{r_{ij}-a\sigma }\right)\exp\left(\frac{\gamma \sigma }{r_{ik}-a\sigma }\right)$$

The summations in Equation (1) are over all neighbors *j* and *k* of atom *i* within a cutoff distance *a*. *A*, *B*, σ, ε, *p*, *q*, λ, h and γ are the potential parameters, which are given in Table 1.

(b) Tersoff potential

The overall potential energy U of the system described by the Tersoff model is given by:

(4) $$U=\frac{1}{2}\sum_{i\neq j}f_{c}\left(r_{ij}\right)\left[f_{R}\left(r_{ij}\right)+b_{ij}f_{A}\left(r_{ij}\right)\right]$$

where $f_{R}\left(r_{ij}\right)=A\exp\left(-\lambda_{1}r_{ij}\right)$ and $f_{A}\left(r_{ij}\right)=-B\exp\left(-\lambda_{2}r_{ij}\right)$ are pair repulsion and attraction functions between atoms *i* and *j*, respectively, and $f_{c}\left(r_{ij}\right)$ is a switching function:

(5) $$f_{c}\left(r_{ij}\right)=\{\begin{array}{ll} 1, & r_{ij}<R\\ \frac{1}{2}+\frac{1}{2}\cos\left[\pi \frac{\left(r_{ij}-R\right)}{\left(S-R\right)}\right], & R<r_{ij}<S\\ 0, & r_{ij}>S \end{array}$$

In Equation (4), the attraction part is modulated by the bond-order term:

(6) $$b_{ij}={\left(1+\beta^{n}\zeta_{ij}^{n}\right)}^{-1/2n}$$

which represents the influence of the local environment on the pair-wise interaction, rendering the Tersoff potential a many-body function. Moreover, the function $\zeta_{ij}$ includes angular contributions based on three-body terms, i.e.,

(7) $$\zeta_{ij}=\sum_{k\neq i,j}f_{c}\left(r_{ik}\right)\omega g\left(\theta_{ijk}\right)$$

where:

(8) $$g\left(\theta_{ijk}\right)=\kappa \left\{1+c^{2}/d^{2}-c^{2}/\left[d^{2}+{\left(h-\cos\theta_{ijk}\right)}^{2}\right]\right\}$$

A, B, $\lambda_{1}$, $\lambda_{2}$, R, S, β, n, ω, κ, c, d and h are given parameters listed in Table 1.

### 2.2. TI for Calculating the Free Energies of Defective Crystals

The thermodynamic integrations were realized based on alchemical pathways by converting a germanium atom (acting as the alchemical atom) to an ideal gas particle [11,12]. Along the path, an atom on the lattice of the perfect crystal was converted into an ideal gas particle for calculating the free energy of the vacancy-containing crystal, while for the interstitial case, the self-interstitial atom was converted into an ideal gas particle. The Gibbs free energies of the crystal containing a vacancy *G^V^* and a self-interstitial *G^I^* at temperature *T* and pressure *P* are given by

(9) $$G^{V}=G^{P}+\int_{0}^{1}\langle \frac{\partial \bar{H}(\lambda )}{\partial \lambda }\rangle d\lambda +k_{B}T\ln\left(\frac{{\langle V\rangle }_{V}}{N\Lambda^{3}}\right)$$

and

(10) $$G^{I}=G^{P}-\int_{0}^{1}\langle \frac{\partial \bar{H}(\lambda )}{\partial \lambda }\rangle d\lambda -k_{B}T\ln\left(\frac{{\langle V\rangle }_{P}}{(N+1)\Lambda^{3}}\right)$$

respectively. *G^P^* is the free energy of the perfect crystal containing *N* atoms, which is calculated through the Einstein crystal method [8]. $\Lambda =h/\sqrt{2\pi mk_{B}T}$ is the thermal de-Broglie wavelength, $k_{B}$ is Boltzmann’s constant, *h* is Plank’s constant, and *m* is the atomic mass. ${\langle V\rangle }_{V}$ and ${\langle V\rangle }_{P}$ are the equilibrium volume of the vacancy-containing system and the perfect crystal system, respectively. $\bar{H}(\lambda )=U(\lambda )+PV(\lambda )$, where U(λ) is the potential energy of the system described by a softcore modification of the original potential function [11,12]. The alchemical pathway was realized by varying λ from 0 to 1. When λ=0, the alchemical atom is a real germanium atom, and it becomes an ideal gas particle when λ=1. The integrand $\frac{\partial \bar{H}(\lambda )}{\partial \lambda }$ in Equations (9) and (10) is given by

(11) $$\frac{\partial \bar{H}(\lambda )}{\partial \lambda }=\frac{\partial U(\lambda )}{\partial \lambda }+P\frac{\partial V(\lambda )}{\partial \lambda }$$

The integration of the second term in Equation (11) is PΔV with ΔV being the volume difference between the systems at the end and beginning of the pathway. It vanishes when the system pressure *P* is 0.

The free energies at other temperatures were calculated using another temperature-dependent thermodynamic integration. The entropy of the system as a function of *T* was calculated according to

(12) $$S(T)=S(T_{m})+{\int_{T_{m}}^{T}\left(\frac{1}{T}\frac{\partial H(T)}{\partial T}\right)}_{P}dT$$

taking the entropy at the melting temperature $S(T_{m})$ as the reference. *H* is the enthalpy of the system, which is equal to the total energy when pressure is 0. The TI in Equation (12) is conducted under constant pressure. The free energies *G*(*T*) at different temperatures are then given by

(13) G(T)=E(T)−TS(T)

where E(T) is the total energy of the system.

### 2.3. Simulation Details

The free energy calculations were based on molecular dynamics (MD) simulations. The simulation boxes were cubic with periodic boundary conditions in the three directions. The atom number *N* was set to 512. The system size effect was checked by varying the atom number from 216 to 1000, and the results showed that 512 atoms were enough for the calculations. All the free energies were calculated at 0 pressure. The temperature and pressure were maintained using the Nosé–Hoover thermostat and barostat implemented in the LAMMPS software package [20]. Twenty-one integrands in Equations (9) and (10) were computed at different λ values evenly distributed between 0 and 1 (additional points were added in the curve at positions with steep slopes). At each λ, 100,000 MD steps were used to equilibrate the system, and more than 5 million steps were used for averaging. The time-step size was set to be 1 fs. The convergence was checked by monitoring the running average values. The integration in Equations (9) and (10) was estimated numerically using the trapezoidal rule. Another series of MD simulations for more than 3 ns were conducted to obtain the total energies and the equilibrium volumes of the systems as a function of temperature. The total energies of the system were fitted to quadratic polynomials and were applied in Equation (12) for the temperature-dependent thermodynamic integrations. 

## 3. Results

**Figure 1:**
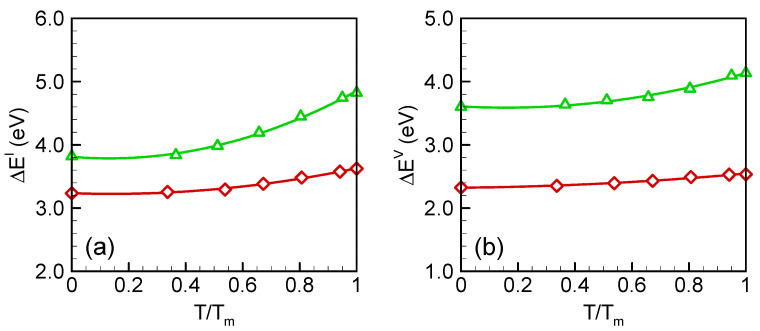
Formation energies of (**a**) self-interstitial (**b**) vacancy in germanium crystal. Red diamond and green delta symbols represent SW and Tersoff potential results, respectively. The lines are quadratic-fitted.

The formation energies $\Delta E^{I,V}$ and formation free energies $\Delta G^{I,V}$ were computed according to

(14) $$\Delta E^{I,V}(T)=E^{I,V}(T)-\frac{N^{I,V}}{N^{P}}E^{P}(T)$$

(15) $$\Delta G^{I,V}(T)=G^{I,V}(T)-\frac{N^{I,V}}{N^{P}}G^{P}(T)$$

where the superscripts *I*, *V* and *P* represent interstitial, vacancy and perfect crystal. The formation energies of self-interstitial and vacancy as a function of temperature are shown in Figure 1a,b, respectively. For both types of point defects, the formation energies increased with temperature, indicating that higher energy configurations were being accessed. The temperature dependence of formation energies predicted by SW is relatively slight in comparison with those by Tersoff. The ground-state formation energies predicted by SW potential were 3.2 eV and 2.3 eV for interstitial and vacancy, respectively, which are very close to the DFT results (3.5 eV for interstitial and 2.6 eV for vacancy) [21,22,23]. At higher temperatures near the melting point, $\Delta E^{I}$ and $\Delta E^{V}$ increased to 3.5 eV and 2.5 eV, respectively, which are in agreement with previous computational results as well as experimental values [3,24]. The Tersoff potential, however, predicted higher formation energies, changing from 3.8 eV to 4.8 eV for interstitial and 3.6 eV to 4.1 eV for vacancy.

**Figure 2:**
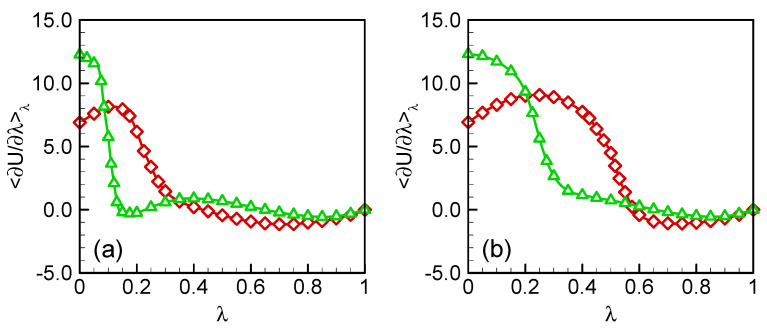
The integrands at different *λ* for (**a**) self-interstitial and (**b**) vacancy. Red for SW and green for Tersoff. The lines are guides for eyes.

The free energies of germanium crystals containing a self-interstitial and a vacancy were calculated, respectively, using thermodynamic integrations based on the alchemical pathways. The integrands in Equations (9) and (10) are shown as a function of *λ* in Figure 2. The integration curves are relatively smooth. There is no divergence occurring at the two ends of the integration.

**Figure 3:**
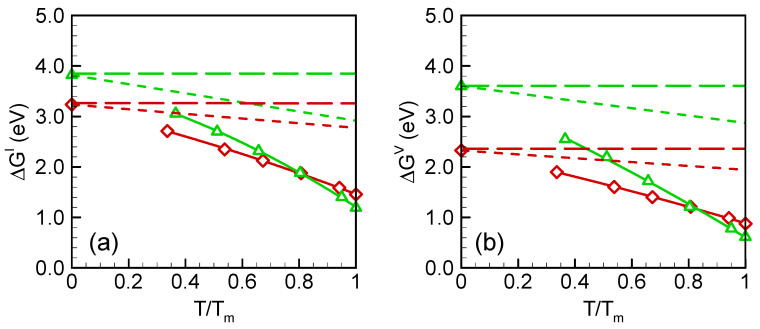
Formation free energies as functions of temperature for (**a**) self-interstitial and (**b**) vacancy. Red and green represent SW and Tersoff results, respectively. The long dashed lines are 0 K formation energies, the short dashed lines are harmonic formation free energies, solid lines are total formation free energies.

The formation free energies as a function of temperature are shown in Figure 3a,b for self-interstitial and vacancy, respectively. In Figure 3, also shown are the 0 K formation energies (long dashed lines) and the harmonic formation free energies (short dashed lines) for comparison. The harmonic formation free energies were calculated using

(16) $$\Delta G_{h}^{I,V}(T)=\Delta E^{I,V}(0)-T\Delta S_{h}^{I,V}$$

where $\Delta S_{h}^{I,V}$ is the harmonic formation entropy. $\Delta S_{h}^{I,V}$ was computed according to

(17) $$\Delta S_{h}^{I,V}=S_{h}^{I,V}\left(T\right)-\frac{N^{I,V}}{N^{P}}S_{h}^{P}\left(T\right)$$

In Equation (17), the harmonic entropies $S_{h}^{I,V}$ and $S_{h}^{P}$ were computed using the formula

(18) $$S_{h}^{X}\left(T\right)=k_{B}\sum_{n=1}^{3N^{X}-3}\left[1-\ln\left(\frac{\hbar \omega_{n}^{X}}{k_{B}T}\right)\right]$$

where $\left\{\omega_{n}^{X}\right\}$ is the normal modes associated with the ground-state structure, X={P,V,I}, and *N^X^* is the system atom number. The normal modes were calculated using the Hessian matrix evaluated from the ground state structure defined as

(19) $$H_{ij}=\frac{\partial^{2}U}{\partial q_{i}\partial q_{j}}$$

where the set $\left\{q_{i}\right\}$ specifies the 3*N^X^* atomic coordinates.

In Figure 3, it is shown that the formation free energies decreased with temperature. For the SW potential (red lines), the harmonic formation free energies are very close to the 0 K formation energies for both interstitial and vacancy. The differences are within 0.5 eV, even near *T_m_*, which indicates that the harmonic entropy only contributes a small part to the formation free energy of point defects. From the perspective of the potential landscape, the inherent structure [25,26] of the ground basins for the perfect crystal and the defective crystals may be very similar. The total formation free energies, however, deviate significantly from the 0 K and harmonic values at a high temperature, decreasing by 1.3 eV for interstitial and 1.1 eV for vacancy at *T_m_* in comparison with harmonic values. For the Tersoff potential, both the harmonic and total formation free energies decreased faster with temperature than those of SW potential. Interestingly, the total formation free energies predicted by the two potentials are very close at melting temperature, ~1.3 eV (on average) for self-interstitial and ~0.75 eV (on average) for vacancy. The point defects formation energies in silicon and germanium at melting temperature are listed in Table 2 and Table 3. The relative deviations were calculated by taking the experimental or DFT formation energies as reference. By comparison, several similarities may be drawn from the values in Table 2 and Table 3. For both silicon and germanium, the deviations of the formation energies predicted by Tersoff are larger than those by SW. However, the deviations of the free energies are pretty close for the two potentials. In comparison with the formation energies, the formation free energies decreased significantly, by approximately 50–70%. The consistency between the two very different potential models increases the confidence of the present results, although it is very hard to evaluate the accuracy of these values due to the rarity of reported results.

The significant decrease in formation free energies indicates that the anharmonic or configurational entropy is increasing dramatically. The harmonic formation entropies calculated based on the ground states for *I* and *V* were 3.6 *k_B_* and 3.0 *k_B_* by SW, and 3.8 *k_B_* and 3.1 *k_B_* by Tersoff, respectively. The consistency between the two potential models is shown again here. We then calculated the total formation entropy based on the formation free energies,

(20) $$\Delta S^{I,V}(T)=\left[\Delta E^{I,V}(T)-\Delta G^{I,V}(T)\right]/T$$

**Figure 4:**
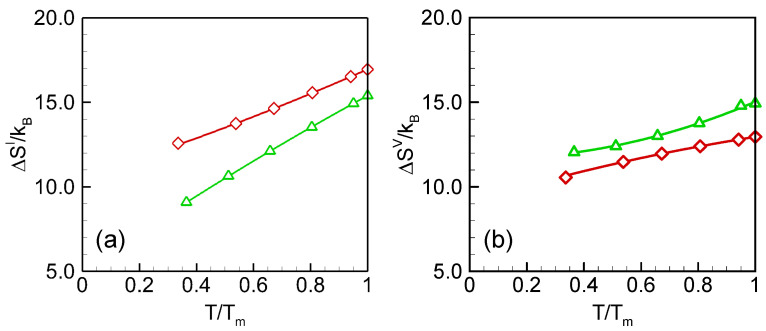
Formation entropies of (**a**) self-interstitial and (**b**) vacancy in germanium crystal. Red and green are results of SW and Tersoff, respectively. The lines are quadratic-fitted.

The formation entropy of self-interstitial and vacancy are shown as functions of temperature in Figure 4. The total formation entropies predicted by the two potentials are also very close at melting temperature, ~15 *k_B_* for self-interstitial and ~14 *k_B_* for vacancy. The harmonic formation entropy accounts for ~20% of the total formation entropy at melting temperature for both point defects. The point defect entropies are relatively less characterized in germanium. Considering the similarity of structure and properties between silicon and germanium, we may make a comparison between the calculated results and those of silicon crystals. Atomic simulations with both electronic structure and empirical methods suggest that the point defect vibrational entropies of formation are significant in silicon crystal: ~4–8 *k_B_* [30,31,32,33,34]. Cowern and coworkers [7] found that the point defects in silicon and germanium crystal transform from “point like” to “extended defects” as the temperature increases. The extended defects have very high configurational entropy in germanium (i.e., 30 *k_B_*). Adding the configurational entropy, it is very possible that the melting point total formation entropy will be comparable to our estimated results.

## 4. Conclusions

In the research, the thermodynamic formation properties of point defects in germanium crystal were calculated using molecular dynamics simulations based on two empirical interatomic potentials, SW and Tersoff. The results were compared with previous research and experimental values wherever possible. The formation energies predicted by both potentials increased slightly with temperature, and the values predicted by SW are in agreement with experimental values, while those by Tersoff are overestimated. The total formation free energies calculated through thermodynamic integrations decreased significantly with temperature due to the increase in entropy. The two potential models showed very good consistencies in the results of total formation free energies and formation entropies at a high temperature. The total formation free energies predicted by the two potentials are very close at melting temperature, ~1.3 eV for self-interstitial and ~0.75 eV for vacancy. The harmonic formation entropies estimated by the two potentials were ~3–4 *k_B_* for both interstitial and vacancy, which are relatively low and account for only ~20% of the total formation entropy, i.e., ~15 *k_B_* at the melting temperature.

Unfortunately, due to the lack of experimental values, it is relatively hard to evaluate the accuracy of the present results. However, the consistency between the results obtained from the different potential models increases the confidence of these calculations. In addition, it should be pointed out that most empirical potential models do not include (or only include the single ground-state configurational) point defect thermodynamic properties during the parameter-fitting procedure. To improve the reliability of MD simulations, it is worthwhile to consider the potential energy landscape more globally in the fitting of an empirical model.

## Figures and Tables

**Table 1:** Empirical potential parameters.

SW		Tersoff	
A	7.049556	A (eV)	1769.0
B	0.602225	B (eV)	491.23
σ (Å)	2.181	$\lambda_{1}$ (Å^−1^)	2.4451
ε (eV)	1.918	$\lambda_{2}$ (Å^−1^)	1.7047
p	4	R (Å)	2.8
q	0	S (Å)	3.1
λ	21	β	9.0166 × 10^−7^
h	0.33333	n	0.75627
γ	1.2	ω	1
a	1.8	κ	1.0
		c	1.0643 × 10^5^
		d	15.652
		h	0.43884

**Table 2:** Formation energies of vacancy in silicon and germanium at *T_m_*.

	Si		Ge	
Exp./DFT ∆*E* (eV)	3.13 ^a^		2.41 ^b^	
	SW	Tersoff	SW	Tersoff
∆*E* (eV)	2.85 ^c^	3.99 ^c^	2.54	4.36
Relative deviation (%)	8.9	27	13	81
∆*G* (eV)	1.11 ^c^	1.06 ^c^	0.88	0.62
Relative deviation (%)	65	66	63	74

^a^ Average of experimental values in Ref. [27] and Ref. [28]; ^b^ Ref. [3] and references therein; ^c^ MD simulation values in Ref. [12].

**Table 3:** Formation energies of interstitial in silicon and germanium at *T_m_*.

	Si		Ge	
Exp./DFT ∆*E* (eV)	4.05 ^d^		3.5 ^e^	
	SW	Tersoff	SW	Tersoff
∆*E* (eV)	4.05 ^c^	4.54 ^c^	3.62	4.82
Relative deviation (%)	0	12	3.4	38
∆*G* (eV)	2.12 ^c^	1.88 ^c^	1.45	1.19
Relative deviation (%)	48	53	58	66

^c^ MD simulation values in Ref. [12].^d^ Ref. [29] and references therein; ^e^ DFT value in Ref. [22].

## Data Availability

All the data that support the findings of this study are available within the article.
